# DNA sequence polymorphism of the *Rhg4* candidate gene conferring resistance to soybean cyst nematode in Chinese domesticated and wild soybeans

**DOI:** 10.1007/s11032-012-9703-1

**Published:** 2012-02-18

**Authors:** Cui-Ping Yuan, Ying-Hui Li, Zhang-Xiong Liu, Rong-Xia Guan, Ru-Zhen Chang, Li-Juan Qiu

**Affiliations:** 1 The National Key Facility for Crop Gene Resources and Genetic Improvement (NFCRI), Institute of Crop Sciences, Chinese Academy of Agricultural Sciences, Beijing, 100081 China; 2 Presently Working at the Biotechnology Research Centre, Jilin Academy of Agricultural Sciences, Changchun, 130033 China

**Keywords:** *Glycine max*, *Glycine soja*, Sequence diversity, *Heterodera glycines*

## Abstract

*Rhg4* is one of the major resistant genes conferring resistance to soybean cyst nematode races 1, 3 and 4. In order to better understand its sequence diversity among different Chinese soybean populations and the impact of human activities on it, we designed 5 primer sets based on its sequence deposited in Genbank (Genbank accession No. AF506518) to obtain the *Rhg4* sequence from 104 Chinese cultivated and wild soybean genotypes, and then analyzed the DNA sequence polymorphism in different Chinese soybean populations. The alignment of *Rhg4* sequence included 5,216 nucleotide base pairs. A total of 67 single nucleotide polymorphisms (SNPs) including 59 single base changes and 8 DNA insertion-deletions (InDels) were identified with a SNP frequency of 1/78. Except for a 14-base InDel, there were 29 SNPs in coding regions, and among them, 13 were non-synonymous (9 in functional domains with 1 in a leucine-rich repeats region, 2 in a transmembrane region and 6 in a Ser/Thr kinase domain). The probability of substitution at each site was not the same, there were two hot spots, one was in the 5′-untranslated region between positions 124 and 804, and the other was in the region between positions 2520 and 3733. Sequence diversity analysis among 104 soybean genotypes showed π = 0.00102 and θ = 0.00218 for *Rhg4*. A domestication bottleneck was found because of lower sequence diversity and 58% unique SNPs loss in landraces compared with *Glycine soja*. Intensive selection increased the sequence diversity of cultivars, which had higher diversity and more unique SNPs than landraces.

**Electronic supplementary material:**

The online version of this article (doi:10.1007/s11032-012-9703-1) contains supplementary material, which is available to authorized users.

## Introduction

Soybean cyst nematode (SCN) (*Heterodera glycines* Ichinohe) is one of the most devastating pathogens in soybean production worldwide and causes substantial yield losses (Li et al. 2011; Wrather et al. 2001; Wrather and Koenning 2009) by feeding on soybean roots, damaging root systems, and reducing the plant’s ability to absorb water and nutrients. Resistant cultivars are considered the best method to control SCN. Many scientists have conducted research programs in an effort to identify resistant sources (Arelli and Wilcox 1997; Arelli et al. 2000; Coordinative group of evaluation of SCN 1993; Lai et al. 2005; Young 1990; Zhang and Dai 1992), identify the genes involved (Caldwell et al. 1960; Lu et al. 2006; Matson and Williams 1965; Rao-Arelli et al. 1992; Vuong et al. 2010; Wang et al. 2001; Winter et al. 2007) or produce resistant varieties (Anand et al. 2004; Diers et al. 2006; Du et al. 2006; Hao et al. 2003; Mengistu et al. 2005; Qiu and Wang 2007; Shannon et al. 2009; Wang et al. 2007). Classical inheritance studies identified five SCN resistance genes in soybean, three recessive genes designated *rhg1*, *rhg2* and *rhg3* were first reported in ‘Peking’ (Caldwell et al. 1960), the dominant gene *Rhg4* was also identified in ‘Peking’, and was linked to the ‘*i*’ locus controlling seed coat color (Matson and Williams 1965), an additional dominant gene, *Rhg5*, was reported in PI 88788 (Rao-Arelli et al. 1992).

Almost 20 years of genetic mapping studies described more than 70 SCN resistance quantitative trait loci (QTLs) (Concibido et al. 2004; Guo et al. 2006; Vuong et al. 2010; Winter et al. 2007; Wu et al. 2009; Yuan et al. 2006). Despite inconsistencies of QTL mapping, it was concluded that *rhg1* and *Rhg4* were two major SCN resistance genes. The *rhg1* locus repeatedly mapped on linkage group (LG) G [chromosome (Chr) 18] in many resistant soybean genotypes (Chang et al. 1997; Concibido et al. 1994, 1997; Guo et al. 2006; Prabhu et al. 1999; Webb et al. 1995; Yue et al. 2001) and provided the greatest level of resistance. Ruben et al. (2006) summarized the construction of integrated physical and genetic maps of a 0.2 cM interval encompassing the *rhg1* locus, and characterized the candidate gene as well as the encoding protein, RHG1, a receptor-like kinase. Li et al.(2009) developed 6 SNP markers based on the variation in *rhg1* and reported their significant improvement of efficiency in marker-assisted selection (MAS) when combined with microsatellite marker BACR-Satt309, although Melito et al. (2010) reported no significant impacts of the LRR-kinase gene on SCN resistance.

Meanwhile *Rhg4* was located on LG A2 (Chr 8) (Chang et al. 1997; Concibido et al. 1994; Guo et al. 2006; Heer et al. 1998; Mahalingam and Skorupska 1995; Webb et al. 1995), 0.35 cM from the *I* locus (Matson and Williams 1965). Several genes associated with stress or defense responses such as chalcone synthase, glucosyl-transferase, heat-shock transcription factor, protein kinase, G10-like protein and restriction fragment length polymorphism molecular marker pBLT65 were close to the *I* and *Rhg4* loci (Heer et al. 1998; Lewers et al. 2002; Matthews et al. 2001; Todd and Vodkin 1996; Webb et al. 1995; Weismann et al. 1992). Two separate research groups isolated the receptor-like kinase candidate gene *Rhg4* from soybean variety ‘Forrest’ by positional cloning (Hauge et al. 2001; Lightfoot and Meksem 2002) and its DNA and protein sequence were lodged in Genbank in 2002 (Genbank accessions AF506518 and AAM44275.1). However, the candidate gene was little studied except for the work of Jang et al. (2004) who reported 3 SNPs and 7 InDels within two regions of *Rhg4* totalling 901 bp by direct sequencing with 2 primer sets.

Like other important crops, soybean has undergone selection by human, involving domestication, intensive breeding, and probable founding events (Gyuhwa and Ram 2008; Hyten et al. 2006). These selection activities likely decrease genetic diversity (Tenaillon et al. 2001; Zhu et al. 2007), change allelic frequencies (Hyten et al. 2006) and eliminate rare alleles (Hyten et al. 2006; Tenaillon et al. 2001). Cultivated soybean (*G. max*) was domesticated from wild soybean (*G*. *soja*) in China (Hymowitz and Newell 1981), and domestication immediately resulted in *G*. *max* landraces (Hyten et al. 2007). Subsequent intensive selection imposed on landraces by soybean breeding created elite soybean cultivars. Hyten et al. (2006) and Yuan et al. (2008) detected effects of domestication bottlenecks in soybeans, but they had inconsistent results regarding intensive selection effects. Hyten et al. (2006) showed that modern soybean breeding had only minimal affects on the allelic structure of the soybean genome, but Yuan et al. (2008) reported an intensive selection bottleneck on *GmHs1* ^*pro*−1^. However, in investigating the founding effects of soybean introduction to North America, they found evidence for only minor and non- significant bottlenecks (Hyten et al. 2006). The cumulative effects of the two genetic bottlenecks caused by founding events and intensive selection led to significant reductions in genetic diversity among North American elite cultivars in comparison with Asian landraces (Hyten et al. 2006).

In the present work we quantified DNA sequence polymorphism of *Rhg4* in Chinese domesticated and wild soybeans by investigating an almost complete *Rhg4* gene sequence in order to better understand its sequence diversity among different Chinese soybean populations and the impact of human activities on the candidate resistance gene. The resulting information may help the development of SNP markers for use in MAS in breeding programs.

## Materials and methods

### Plant materials

The plant materials were selected from 27 provinces (autonomous regions or municipalities) of China (MOESM1) and represent three populations (*G*. *soja*, landraces and cultivars). The population of *G*. *soja* consisted of 28 accessions from 14 provinces (autonomous regions), landraces were represented by 51 accessions from 22 provinces (autonomous regions or municipalities), cultivars were from 7 provinces (municipalities), including 8 soybean genotypes from our core collection resistant to SCN (Ma et al. 2006), 3 awarded varieties, 4 parental lines (varieties) of our soybean genetic populations, and 10 elite cultivars. Genomic DNA was extracted from seedlings of wild accessions and from seed of domesticated genotypes as described by Yuan et al. (2008).

### Primers design and polymerase chain reaction (PCR) amplification

Five pairs of primers (MOESM2) were designed from the sequence of the *Glycine max* receptor-like kinase *Rhg4* gene (GenBank accession AF506518), with overlaps of 137–218 bp between adjacent PCR regions. PCR was carried out in total volumes of 20 μl consisting of 60 ng DNA, 1× PCR TaKaRa Buffer, 0.15 μM of each primer, 0.15 mM dNTP and 0.5 U TaKaRa ExTaq polymerase. PCR amplification conditions were as described by Yuan et al. (2008).

### Sequencing

PCR products were separated on 1.0% agarose gels stained with ethidium bromide. The PCR primer set of 3561U22 and 4782L26 produced 3 amplicons with about 90 nucleotide base differences in length. A pre-experiment determined that the smallest amplicon was the most similar to AF506518 and located in LG A2 (Chr 8) by blastn against AF506518 (unshown), so we chose the smallest amplicon as a target fragment. The fragment was purified using a DNA fragment purification kit (Biotech), cloned into the pMD18-T vector (TaKaRa) and then sequenced with primers of M13F and M13R. The other 4 PCR primer sets each produced single amplicons, and the PCR products were directly sequenced with PCR primers after being collected and purified. When necessary, sequencing primers (MOESM2) were designed from the sequencing information to assure accuracy of sequence determination.

### Sequence analysis

Sequences were assembled with the SeqMan tool of DNAstar software, and the sequence of cv. Huipizhiheidou (HPZhHD) was interrogated by BLAST searches in Genbank for identity confirmation. Sequence alignment was performed using Clustalx 1.8 with manual refinement. Single base changes and single or multiple base InDels were collectively preferred as SNPs. Only informative SNP sites were selected to build haplotypes. Differential regions of the DNA sequence were predicted and located following the method of Tang and Lewontin (1999). Two DNA polymorphism measures of nucleotide diversity (π and θ) and haplotype diversity were calculated with DnaSP v.5.10.01 software. A neighbor-joining phyolgenetic tree was constructed using MEGA 5.0 software with a Kimura 2-parameter model and 1,000 bootstrap replications.

## Results

### Sequence comparison of *Rhg4* with AF506518

Sequence assembly with the SeqMan program generated a continous sequence of 5,216 bp and a BLAST search showed it had 98% identity at the nucleotide level with the receptor-like kinase *Rhg4* gene (AF506518), therefore the sequence was presumed to be *Rhg4*. Alignment with AF506518, there were 49 base changes, 2 single-base inserts and one three-base insert (MOESM3). Both single-base inserts occurred in the 5′-untranslated region (UTR), whereas the three-base insert was in the second exon, but it was not a frame-shift mutation. The predicated protein therefore had one more amino acid than the receptor-like kinase RHG4 (AAM44275.1).

### DNA variants of *Rhg4*

We obtained the 5,216 bp DNA sequence of *Rhg4* from 104 soybean genotypes, representing members of the three distinct populations, viz. *G*. *soja*, landraces and cultivars. Surprisingly high sequence polymorphism was found in *Rhg4*, a total of 67 SNPs, including 59 single base changes and 8 DNA InDels, were identified with an SNP frequency of 1/78 (Table 1). Five InDel loci had 3 alleles each, and a 14-base gap occurred in the coding region of one wild soybean genotype presumably leading to a frame-shift mutation. Except for the 14-base Indel, there were 29 SNPs in coding regions, among them 13 were non-synonymous and 16 were synonymous. Of the 59 single base changes, 40 were involved in transitions, and 19 were transversions with a transition:transversion ratio of 2:1.

**Table 1:** Numbers of SNPs in *Rhg4* among 104 soybean genotypes

	5′-UTR	Coding region	Intron	3′-UTR	Total
InDels	6	1	1	0	8
Single base changes					
Transitions	6	22	12	0	40
Transversions	6	7	4	2	19
Total	12	29	16	2	59
Total	18	30	17	2	67

The predicted RHG4 protein consisted of 894 amino acids, characterized with 3 functional domains of 6 extracellular leucine-rich repeats (LRRs) (56–401), a transmembrane (TM) domain (449–471) and an intracellular Ser/Thr kinase (STK) domain (544–820). Of the 13 non-synonymous nucleotide substitutions, 9 occurred in functional domains, with 1 in the LRR region, 2 in the TM region and 6 in the STK domain.

The probability of substitution at each site in *Rhg4* was not the same, there were two hot spots with higher probabilities of substitution. One hot spot occurred in the 5′-UTR region between positions 124 and 804 and the other was in the region between positions 2,520 and 3,733.

### DNA polymorphism of *Rhg4* among the three soybean populations

**Fig. 1:**
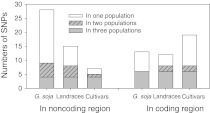
Number of shared and unique SNPs in the three soybean populations

Unique and shared SNPs among the three soybean populations were investigated (Fig. 1). A total of 41 SNPs were detected in *G*. *soja*, of which 26 were unique and not found in the two *G*. *max* populations. Landraces contained 27 SNPs, of which 11 were unique. Cultivars also had 26 SNPs, 13 of which were unique. While examining the coding region of *Rhg4*, we found that cultivars had the largest number of sequence variants with 19 SNPs, 11of which were unique; however *G*. *soja* and landraces had 13 and 12 SNPs, with only 7 and 4, respectively being unique.

Nucleotide diversity analysis on 104 soybean genotypes showed π = 0.00102 and θ = 0.00218 for *Rhg4* (Table 2). Among the three populations, *G*. *soja* had the highest diversity with π = 0.00114 and θ = 0.00164, followed by cultivars, and landraces had the lowest with π = 0.00090 and θ = 0.00098. Sequence diversity in different regions of the gene showed obvious differences, the highest sequence diversity occurred in synonymous sites of the coding region, followed by intron, the 5′-UTR region, and in non-synonymous sites of the coding region, with the lowest sequence diversity in the 3′-UTR. Cultivars had the highest sequence diversity in the coding region among the three populations.

**Table 2:** Nucleotide diversity for *Rhg4* (× 10^3^) in the soybean populations

Population	5′-UTR		Coding region						Intron		3′-UTR		Total	
	π	θ	π(sny)	θ(sny)	π(Nsny)	θ(Nsny)	π(total)	θ(total)	π	θ	π	θ	π	θ
*G*. *max*														
Landraces	0.91	0.96	2.49	2.41	0.69	0.55	1.12	0.99	0.89	1.87	0.00	0.00	0.90	0.98
Cultivars	0.86	0.46	3.33	4.11	0.89	1.17	1.48	1.88	0.86	1.11	0.00	0.00	1.07	1.22
Total	0.88	0.88	2.74	3.80	0.75	1.10	1.22	1.75	0.87	2.29	0.00	0.00	0.95	1.41
*G*. *soja*	1.31	2.24	2.82	2.81	0.71	0.63	1.21	1.16	1.47	3.24	0.22	0.78	1.14	1.64
Total	1.05	2.01	2.79	4.79	0.75	1.23	1.24	2.08	1.04	4.30	0.06	0.58	1.02	2.18

Three haplotypes of *Rhg4* commonly occurred among the three soybean populations, the other 23 haplotypes were in only one or two of the populations. Among the 23 haplotypes, 14 were uniquely detected in *G*. *soja*, 5 were uniquely in cultivars, 3 were uniquely in landraces, and 1 was detected in both cultivars and landraces. As for haplotype diversity, *G*. *soja* had the highest haplotype diversity at 0.97, whereas cultivars and landraces had lower values (0.89 and 0.80, respectively).

## Discussion

The SNP frequency in *Rhg4* among the 104 soybean accessions was 1/78, obviously higher than earlier estimates of 1/106 (Yuan et al. 2008), 1/107 (Hyten et al. 2006), 1/273 (Zhu et al. 2003) and 1/343 (Van et al. 2005) in soybean. When compared with other plants, the SNP frequency was lower than those reported in maize (Ching et al. 2002; Tenaillon et al. 2001; Yamasaki et al. 2005) and chickpea (Rajesh and Muehlbauer 2008), and higher than those in bread wheat (Ravel et al. 2007) and *Arabidopsis thaliana* (Schmid et al. 2003; Clark et al. 2007). The difference for SNP frequencies maybe due to different samples and different genomic regions. Although higher SNP frequency was detected than other reports for soybean, sequence diversity among the 3 soybean populations was obviously low compared with other reports among corresponding soybean populations (Hyten et al. 2006). The reason probably was that soybean resources we used were only from China, having somewhat narrower genetic variation.

As for nucleotide mutation type, there is a clear transition bias probable due of the high spontaneous rate of deamination of 5-methl cytosine to thymidine in the CpG dinucleotides (Vignal et al. 2002), that is, GC content is probably linked to the ratio of transitions to transversions. In our study the ratio was 2.1, similar to 2.12 reported in soybean by Van et al. (2005), 2 reported in humans (Wang et al. 1998) and mouse (Lindblad-Toh et al. 2000), and relatively greater than 1.3 reported in soybean (Yuan et al. 2008), but was contrary to 0.93 reported by Zhu et al. (2003) in soybean. The difference among different species and even among different samples in the same species was probably related with GC content in observed genomic region. Of course some other factors leading to DNA mutation may influence the ratio.

Domestication represents the first result of human selection in soybean. Hyten et al. (2006) reported that landraces retained only 66% (π) and 49% (θ) of the nucleotide diversity found in *G*. *soja*, and had lost 81% of the rare alleles in *G*. *soja*, thus representing a domestication bottleneck. Considering the overall sequence region of *Rhg4*, we also found a domestication bottleneck because firstly the sequence diversity within landraces was obviously low (π = 0.00090 and θ = 0.00098) compared with *G*. *soja* (π = 0.00114 and θ = 0.00164) (Table 2), and secondly, landraces lacked 58% of the unique sequence variants present in *G*. *soja* despite that 2 novel SNPs happened (Fig. 1). However, the domestication bottleneck was slighter than that reported by Hyten et al. (2006). It maybe contributed to frequently communication among agricultural people since the Shang Dynasty or earlier and several domestication centers in China (Hymowitz and Newell 1981, Hymowitz 1970).

Intensive selection imposed in modern soybean breeding programs is generally thought to reduce genetic diversity of elite soybean cultivars (Gizlice and Burton 1994; Miranda et al. 2001; Concibido et al. 2004; Xiong et al. 2008), but Hyten et al. (2006) failed to find large effects of intensive selection based on DNA sequence diversity. In the present study, a clearly higher sequence diversity was found within cultivars (π = 0.00107 and θ = 0.00122) than in landraces (π = 0.00090 and θ = 0.00098), especially for the sequence diversity in the coding region cultivars had sequence diversity increases of π = 0.00036 and θ = 0.00089 compared to landraces (Table 2). In regard to unique SNPs, cultivars had two more than landraces in the overall sequence region, but seven more in the coding region (Fig. 1). This suggested that intensive selection has increased the sequence diversity for *Rhg4* in cultivars. The reason was probably the effect of selection in breeding programs on SCN disease, or other traits, such as oil content (*Oil 1*-*1*), protein content (*Prot 17*-*4*) and seed weight (*Sd wt 4*-*5*) associated with yield, whose loci were close to *Rhg4* (http://soybase.org/MarkerDB/MapFeatureSearch.php?OutPutType=HTML&mapset=GmComposite2003&MapName=A2&FeatureType=All_Types&FeatureStart=0&FeatureStop=9999). Studies on association of *Rhg4* alleles with SCN resistance, and on association mapping of SCN disease, yield and seed quality traits mentioned above on LG A2 (Chr 8) maybe helpful for explanation the effect of intensive selection on *Rhg4* sequence diversity. In addition, incorporation of exotic germplasm from USA, France and Japan into breeding program (MOESM1) may also contribute to the high sequence diversity in cultivars.

Although there were pedigree relationships among some soybean elite varieties or lines in the population of cultivars (MOESM1), which may decrease the sequence diversity, DNA variations were detected among them (MOESM4), indicating possible different allele origins or recombination occurrence. For example, the four cultivars of Hefeng 25, Hefeng 23, Jilin 47, and Suinong 14 were pedigree, but they were grouped into two clusters. Similar case also happened among the Kangxianchong 1, Kangxianchong 2 and Kangxianchong 3.

## Electronic supplementary material

Below is the link to the electronic supplementary material.

Soybean genotypes used for cloning and sequence diversity analysis of *Rhg4* (PDF 36 kb)

Primers designed for PCR amplification and sequencing (PDF 36 kb)

DNA Sequence alignment of *Rhg4* between HPZhHD and AF506518 (PDF 735 kb)

Neighbor-joining phylogenetic tree of 25 soybean varieties from the analysis of the alignment of *Rhg4* (PDF 20 kb)

## References

[CR1] Anand SC, Shannon JG, Wrather JA, Arelli PR, Sleper DA, Young LD (2004). Registration of S97-1688 soybean germplasm line high in protein content and resistant to soybean cyst nematode. Crop Sci.

[CR2] Arelli AP, Wilcox JA (1997). Soybean germplasm resistant to races 1 and 2 of *Heterodera glycines*. Crop Sci.

[CR3] Arelli PR, Sleper DA, Yue P, Wilcox JA (2000). Soybean reaction to races 1 and 2 of *Heterodera glycines*. Crop Sci.

[CR4] Caldwell BE, Brim CA, Ross JP (1960). Inheritance of resistance of soybeans to the cyst nematode, *Heterodera glycines*. Agron J.

[CR5] Chang SJC, Doubler TW, Kilo VY, Abu-Thredeih J, Prabhu R, Freire V, Suttner R, Klein J, Schmidt ME, Gibson PT, Lightfoot DA (1997). Association of loci underlying field resistance to soybean sudden death syndrome (SDS) and cyst nematode (SCN) race 3. Crop Sci.

[CR6] Ching A, Caldwell KS, Jung M, Dolan M, Smith OS, Tingey S, Morgante M, Rafalski AJ (2002). SNP frequency, haplotype structure and linkage disequilibrium in elite maize inbred lines. BMC Genetics.

[CR7] Clark RM, Schweikert G, Toomajian C, Ossowski S, Zeller G, Shinn P, Warthmann N, Hu TT, Fu G, Hinds DA (2007). Common sequence polymorphisms shaping genetic diversity in *Arabidopsis thaliana*. Science.

[CR8] Concibido VC, Denny RL, Boutin SR, Hautea R, Orf JH, Young ND (1994). DNA marker analysis of loci underlying resistance to soybean cyst nematode (*Heterodera glycines* Ichinohe). Crop Sci.

[CR9] Concibido VC, Lange DA, Denny RL, Orf JH, Young ND (1997). Genome mapping of soybean cyst nematode resistance genes in Peking, PI 90763, and PI 88788 using DNA markers. Crop Sci.

[CR10] Concibido VC, Diers BW, Arelli PR (2004). A decade of QTL mapping for cyst nematode resistance in soybean. Crop Sci.

[CR11] Coordinative Group of Evaluation of SCN (1993). Evaluation of soybean germplasm for resistance to race 1, 3 and 4 of the soybean cyst nematode. Soybean Sci.

[CR12] Diers BW, Cary TR, Thomas DJ, Nickell CD (2006). Registration of ‘LD00-3309’soybean. Crop Sci.

[CR13] Du ZQ, Tian ZY, Gao GJ, Zhou CJ, Wang MZ, Li ZX, Wu YK, Li XB (2006). Application and problems of using resistant varieties to SCN in Heilongjiang Province. Heilongjiang Agric Sci.

[CR14] Gizlice ZC, Burton TE (1994). Genetic base for North American public soybean cultivars released between 1947 and 1988. Crop Sci.

[CR15] Guo B, Sleper DA, Nguyen HT, Arelli PR, Shannon JG (2006). Quantitative trait loci underlying resistance to three soybean cyst nematode populations in soybean PI 404198A. Crop Sci.

[CR16] Gyuhwa C, Ram JS (2008). Broadening the genetic base of soybean: a multidisciplinary approach. Crit Rev Plant Sci.

[CR17] Hao XX, Jiang HL, Xu R, Wang JC (2003). Qihuang 28, a soybean variety with high oil content and resistance to soybean cyst nematode. Soybean Bull.

[CR18] Hauge BM, Wang ML, Parsons JD, Parnell LD (2001) Nucleic acid molecules and other molecules associated with soybean cyst nematode resistance. U.S. Pat Appl Publ No. 20030005491

[CR19] Heer JA, Knap HT, Mahalingam R, Shipe ER, Arelli PR, Matthews BF (1998). Molecular markers for resistance to *Heterodera glycines* in advanced soybean germplasm. Mol Breed.

[CR20] Hymowitz T (1970). On the domestication of the soybean. Econ Bot.

[CR21] Hymowitz T, Newell CA (1981). Taxonomy of the genus *Glycine*, domestication and uses of soybeans. Econ Bot.

[CR22] Hyten DL, Song QJ, Zhu YL, Choi IY, Nelson RL, Costa JM (2006). Impacts of genetic bottlenecks on soybean genome diversity. Proc Natl Acad Sci USA.

[CR23] Hyten DL, Choi IY, Song Q, Shoemaker RC, Nelson RL, Costa JM, Specht JE, Cregan PB (2007). Highly variable patterns of linkage disequilibrium in multiple soybean populations. Genetics.

[CR24] Jang S, Van K, Kim MY, Gwag J, Jang H, Lee S (2004). SNP discovery and mapping of a major gene *Rhg4* conferring resistance to soybean cyst nematode. Korean J Breed.

[CR25] Lai YC, Lin H, Fang WC, Yao ZC, Qi N, Wang QX, Yang XF, Li H (2005). The excellent wild soybean resource screening, evaluation and utilization in Heilongjiang. China Agric Sci Bull.

[CR26] Lewers K, Heinz R, Beard H, Marek L, Matthews B (2002). A physical map of a gene-dense region in soybean linkage group A2 near the black seed coat and *Rhg4* loci. Theor Appl Genet.

[CR27] Li YH, Zhang C, Gao ZS, Smulders MJM, Ma Z, Liu ZX, Nan HY, Chang RZ (2009). Development of SNP markers and haplotype analysis of the candidate gene for *rhg1*, which confers resistance to soybean cyst nematode in soybean. Mol Breed.

[CR28] Li Y, Qi X, Chang R, Qiu L, Sudaric A (2011). Evaluation and utilization of soybean fermplasm for resistance to cyst nematode in China. Soybean-molecular aspects of breeding.

[CR29] Lightfoot DA, Meksem K (2002) Isolated polynucleotides and polypeptides relating to loci underlying resistance to soybean cyst nematode and soybean sudden death syndrome and methods employing same. U.S. Pat Appl Publ No. 2002144310

[CR30] Lindblad-Toh K, Winchester E, Daly MJ, Wang DG, Hirschhorn JN, Laviolette JP, Ardlie K, Reich DE, Robinson E, Sklar P (2000). Large-scale discovery and genotyping of single-nucleotide polymorphisms in the mouse. Nat Genet.

[CR31] Lu WG, Gai JY, Zheng YZ, Li WD (2006). Construction of a soybean genetic linkage map and mapping QTLs resistant to soybean cyst nematode (*Heterodera glycines* Ichinohe). Acta Agron Sin.

[CR32] Ma Y, Wang W, Wang L, Ma F, Wang P, Chang R, Qiu L (2006). Genetic diversity of soybean and the establishment of a core collection focused on resistance to soybean cyst nematode. J Integr Plant Biol.

[CR33] Mahalingam R, Skorupska HT (1995). DNA markers for resistance to *Heterodera glycines* I. Race 3 in soybean cultivar Peking. Breed Sci.

[CR34] Matson AL, Williams LF (1965). Evidence of a fourth gene for resistance to the soybean cyst nematode. Crop Sci.

[CR35] Matthews BF, Devine TE, Weisemann JM, Beard HS, Lewers KS, MacDonald MH, Park Y, Maiti R, Lin J, Kuo J, Pedroni MJ, Cregan PB, Saunders JA (2001). Incorporation of sequenced cDNA and genomic markers into the soybean genetic map. Crop Sci.

[CR36] Melito S, Heuberger AL, Cook D, Diers B, MacGuidwin AE, Bent AF (2010) A nematode demographics assay in transgenic roots reveals no significant impacts of the *Rhg 1* locus LRR-kinase on soybean cyst nematode resistance. BMC Plant Biol 10:104. http://www.biomedcentral.com/1471-2229/10/104 10.1186/1471-2229-10-104 PMC3095272 20529370

[CR37] Mengistu A, Kilen TC, Donald PA (2005). Registration of D98-1218 soybean germplasm line resistant to phytophthora rot (Rps2) and soybean cyst nematode races 3 (HG Type 0) and 14 (HG Type 1.3.6.7). Crop Sci.

[CR38] Miranda GV, Sediyama CS, Reis MS, Cruz CD (2001). Genetic diversity among elite Brazilian soybean cultivars with narrow genetic base. Crop Breed Appl Biotech.

[CR39] Prabhu PR, Njiti VN, Bell-Johnson B, Johnson JE, Schmidt ME, Klein HJ, Lightfoot DA (1999). Selecting soybean cultivars for dual resistance to soybean cyst nematode and sudden death syndrome using two DNA markers. Crop Sci.

[CR40] Qiu LJ, Wang SM (2007). Characteristcs of Chinese soybean varieties released in 1993–2004.

[CR41] Rajesh PN, Muehlbauer FJ (2008). Discovery and detection of single nucleotide polymorphism (SNP) in coding and genomic sequences in chickpea (*Cicer arietinum* L.). Euphytica.

[CR42] Rao-Arelli AP, Anand SC, Wrather JA (1992). Soybean resistance to soybean cyst nematode race 3 is conditioned by an additional dominant gene. Crop Sci.

[CR43] Ravel C, Praud S, Canaguier A, Dufour P, Giancola S, Balfourier F, Chalhoub B, Brunel D, Linossier L, Dardevet M, Beckert M, Rousset M, Murigneux A, Charmet G (2007). DNA sequence polymorphisms and their application to bread wheat quality. Euphytica.

[CR44] Ruben E, Jamai A, Afzal J, Njiti VN, Triwitayakorn K, Iqbal MJ, Yaegashi S, Bashir R, Kazi S, Arelli P, Town CD, Ishihara H, Meksem K, Lightfoot DA (2006). Genomic analysis of the *rhg1* locus: candidate genes that underlie soybean resistance to the cyst nematode. Mol Genet Genomics.

[CR45] Schmid KJ, Sörensen TR, Stracke R, Törjék O, Altmann T, Mitchell-Olds T, Weisshaar B (2003). Large-scale identification and analysis of genome-wide single-nucleotide polymorphisms for mapping in *Arabidopsis thaliana*. Genome Res.

[CR46] Shannon JG, Lee J, Wrather JA, Sleper DA, Rouf Mian MA, Bond JP, Robbins RT (2009). Registration of S99-2281 soybean germplasm line with resistance to frogeye leaf spot and three nematode species. J Plant Regist.

[CR47] Tang H, Lewontin RC (1999). Locating regions of differential variability in DNA and protein sequences. Genetics.

[CR48] Tenaillon M, Sawkins MC, Long AD, Gaut RL, Doebley JF, Gaut BS (2001). Pattern of DNA sequence polymorphism along chromosome 1 of maize (*Zea mays* ssp. mays L.). Proc Natl Acad Sci USA.

[CR49] Todd JJ, Vodkin LO (1996). Duplications that suppress and deletions that restore expression from a chalcone synthase multigene family. Plant Cell.

[CR50] Van K, Hwang EY, Kim MY, Park HJ, Lee SH, Cregan PB (2005). Discovery of SNPs in soybean genotypes frequently used as the parents of mapping populations in the United States and Korea. J Hered.

[CR51] Vignal A, Milan D, SanCristobal M, Eggen A (2002). A review on SNP and other types of molecular markers and their use in animal genetics. Genet Sel Evol.

[CR52] Vuong TD, Sleper DA, Shannon JG, Nguyen HT (2010). Novel quantitative trait loci for broad-based resistance to soybean cyst nematode (*Heterodera glycines* Ichinohe) in soybean PI 567516C. Theor Appl Genet.

[CR53] Wang DG, Fan JB, Siao CJ, Berno A, Young P, Sapolsky R, Ghandour G, Perkins N, Winchester E, Spencer J (1998). Large-scale identification, mapping, and genotyping of single-nucleotide polymorphisms in the human genome. Science.

[CR54] Wang D, Arelli PR, Shoemaker RC, Diers BW (2001). Loci underlying resistance to race 3 of soybean cyst nematode in *Glycine soja* plant introduction 468916. Theor Appl Genet.

[CR55] Wang LX, Wang SY, Wang SR, Yuan M, Han DW, Wang F, Li XM (2007). Character and application of soybean variety Nenfeng No. 18 with high oil and resistant to cyst nematode. Heilongjiang Agric Sci.

[CR56] Webb DM, Baltazar BM, Rao-Arelli AP, Schupp J, Clayton K, Keim P, Deavis WD (1995). Genetic mapping of soybean cyst nematode race-3 resistance loci in the soybean PI437654. Theor Appl Genet.

[CR57] Weismann JM, Matthews BF, Devine TE (1992). Molecular markers located proximal to the soybean cyst nematode resistance gene, *Rhg4*. Theor Appl Genet.

[CR58] Winter SMJ, Shelp BJ, Anderson TR, Welacky TW, Rajcan I (2007). QTL associated with horizontal resistance to soybean cyst nematode in *Glycine soja* PI464925B. Theor Appl Genet.

[CR59] Wrather JA, Koenning SR (2009). Effects of diseases on soybean yields in the United States 1996 to 2007. Plant Health Progress.

[CR60] Wrather JA, Anderson TR, Arsyad DM, Tan Y, Ploper LD, Porta-Puglia A, Ram HH, Yorinori JT (2001). Soybean disease loss estimates for the top ten soybean-producing countries in 1998. Can J Plant Pathol.

[CR61] Wu X, Blake S, Sleper DA, Shannon JG, Cregan P, Nguyen HT (2009). QTL, additive and epistatic effects for SCN resistance in PI 437654. Theor Appl Genet.

[CR62] Xiong DJ, Zhao TJ, Gai JY (2008). Parental analysis of soybean cultivars released in China. Scientia Agric Sin.

[CR63] Yamasaki M, Tenaillon MI, Bi IV, Schroederr SG, Sanchez-Willeda H, Doebley JF, Gaut BS, McMullen MD (2005). A large-scale screen for artificial selection in maize identifies candidate agronomic loci for domestication and crop improvement. Plant Cell.

[CR64] Young LD (1990). Soybean germplasm evaluated for resistance to races 3, 5, and 14 of soybean cyst nematode. Crop Sci.

[CR65] Yuan CP, Chang RZ, Qiu LQ (2006). Progress on genetic mapping and gene cloning of cyst nematode resistance in soybean. Chin Bull Bot.

[CR66] Yuan C, Zhou G, Li Y, Wang K, Wang Z, Li X, Chang R, Qiu L (2008). Cloning and sequence diversity analysis of *GmHs1*^*pro*−*1*^ in Chinese domesticated and wild soybeans. Mol Breed.

[CR67] Yue P, Sleper DA, Arelli PR (2001). Mapping resistance to multiple races of *Heterodera glycines* in soybean PI89772. Crop Sci.

[CR68] Zhang L, Dai Q (1992). Study on identification of soybean germplasm resistant to race 5 of soybean cyst nematode. Soybean Sci.

[CR69] Zhu YL, Song QJ, Hyten DL, Van Tassell CP, Matukalli LK, Grimm DR, Hyatt SM, Fickus EW, Young ND, Cregan PB (2003). Single-nucleotide polymorphisms in soybean. Genetics.

[CR70] Zhu Q, Zheng X, Luo J, Gaut BS, Ge S (2007). Multilocus analysis of nucleotide variation of *Oryza sativa* and its wild relatives: Severe bottleneck during domestication of rice. Mol Biol Evol.

