# Supplementary material for: DNA sequence polymorphism of the Rhg4 candidate gene conferring resistance to soybean cyst nematode in Chinese domesticated and wild soybeans
Source: Mol Breed. 2012 Feb 18;30(2):1155–62. doi: 10.1007/s11032-012-9703-1 (PMC3410032; doi:10.1007/s11032-012-9703-1)
Supplement: Supplementary file 1 — Soybean genotypes used for cloning and sequence diversity analysis of Rhg4 (PDF 36 kb) [file 11032_2012_9703_MOESM1_ESM.pdf]

DNA sequence polymorphism of the *Rhg4* candidate gene conferring resistance to soybean cyst nematode in Chinese domesticated and wild soybeans

Moleculare Breeding

Cuiping Yuan · Yinghui Li · Zhangxiong Liu · Rongxia Guan · Ruzhen Chang · Lijuan Qiu
Corresponding author, E-mail: qiu_lijuan@263.net

MOESM1 Soybean genotypes used for cloning and sequence diversity analysis of *Rhg4*

**Name**	**Accession**	**Province**	**Longitude (° E)**	**Latitude (° N)**	**Population**	**Predigree**
Zaoshu 18	ZDD18396	Beijing	116.5	39.9	Cultivar	7902 (Youbian 30 × Clark 63 [a]) × 7821 (Naiyinheidou × Youbian 31)
Zhongdou 28	ZDD23892	Hubei	114.2	30.4	Cultivar	Ludou 4 × L 83-4387 [a]
Dongnong 47-1C	ZDD17713	Heilongjiang	126.4	45.4	Cultivar	Keshuang × Jizaoshengqingbaidou [b]
Hefeng 23	ZDD06821	Heilongjiang	105.5	28.5	Cultivar	Xiaolidou 9 × Fengshou10
Hefeng 25	ZDD06823	Heilongjiang	105.5	28.5	Cultivar	Hefeng 23 × Ke 4430-20 (Ke 69-5236 × Tokachi-Nagaha [b])
Heinong 47	-	Heilongjiang	126.4	45.4	Cultivar	Heinong 40 × Ha 92-2463
Kangxianchong 1	ZDD22649	Heilongjiang	125.2	46.2	Cultivar	Fengshou 12 × Franklin [a]
Kangxianchong 2	ZDD22650	Heilongjiang	125.2	46.2	Cultivar	Nenfeng 9 × (Nenfeng 10 × Franklin [a])
Kangxianchong 3	-	Heilongjiang	125.2	46.2	Cultivar	Kangxianchong 2 × 8314-122
Nenfeng 15	ZDD22652	Heilongjiang	125.2	49.2	Cultivar	CN 210 [a] × Heihe 3
Suiheidadou	-	Heilongjiang	126.6	46.4	Cultivar	Selected from landrace of Heidou
Suinong 14	ZDD22648	Heilongjiang	126.6	46.4	Cultivar	Hefeng 25 × Suinong 8[Suinong 4 × (Sui 77-5047 × Amsoy [a])]
You 1298	ZDD20449	Hubei	114.2	30.4	Cultivar	Williams [a] × 82-16
You 232	ZDD20358	Hubei	114.2	30.4	Cultivar	82-876 × Evans [a]
You 368	ZDD23586	Hubei	114.2	30.4	Cultivar	Yuejino 5 × Suwon 197
Jihuang 138	ZDD22798	Jilin	124.5	43.3	Cultivar	(Changnong 4 × Jilin 21) × Clenson [c]
Jilin 47	ZDD23713	Jilin	124.5	43.3	Cultivar	8403-74 × [Hefeng 25 × (Jilin 20 × Ludou 4)]
Tongnong 13	ZDD23792	Jilin	126	41.5	Cultivar	Tongjiao 86-959 × Changnong 4
Tie 86142-18	ZDD18095	Liaoning	123.9	42.3	Cultivar	Tie 78012-5-3[Tiefeng 18 × (6308-9-1 × Tokachi-Nagaha [b])] × Tie 8114-7-3
Wei J127	ZDD23221	Shandong	119.1	36.7	Cultivar	Ji 81-155 × Weifuxuan
Jin 1267	-	Shanxi	112.5	37.9	Cultivar	untraceble
Jinpin 82	-	Shanxi	112.5	37.9	Cultivar	Selected from PI 88788 [a]
Jindou 11	ZDD18843	Shanxi	112.5	37.9	Cultivar	Selected from a mutant of cv. Mohe 1

MOESM1 Soybean genotypes used for cloning and sequence diversity analysis of *Rhg4* （Continued）

**Name**	**Accession**	**Province**	**Longitude (° E)**	**Latitude (° N)**	**Population**	**Predigree**
Jinpin 42 (JP42)	ZDD23089	Shanxi	112.5	37.9	Cultivar	Selected from a recurrent population
Jinpin 78 (JP78)	ZDD23177	Shanxi	112.5	37.9	Cultivar	Jinda501×Yingxianxiaoheidou
Dangshangdajianke	ZDD11401	Anhui	116.3	34.4	Landrace	
Chadou	ZDD10058	Shandong	117.5	37.5	Landrace	
Pingdinghei	ZDD03026	Shandong	117.1	36.2	Landrace	
Tongxianhuangdou	ZDD07993	Beijing	116.7	39.9	Landrace	
Shaxianwudou	ZDD06438	Fujian	117.8	26.4	Landrace	
Heidouzi	ZDD16820	Guangdong	111.3	22.5	Landrace	
Luodingqingdou	ZDD16775	Guangdong	111.3	22.5	Landrace	
Gongchengqingpidou	ZDD06814	Guangxi	110.8	24.9	Landrace	
Huanjiangbayuehuang	ZDD17042	Guangxi	108.3	24.8	Landrace	
Yaxianhuangdou	ZDD16859	Hainan	109.3	18.1	Landrace	
Yazhouheidou	ZDD16840	Hainan	109.3	18.1	Landrace	
Daheidou	ZDD08510	Hebei	114.8	37.4	Landrace	
Daheidou	ZDD08488	Hebei	115.5	39	Landrace	
Mangzhangzixiaoliheidou	ZDD18512	Hebei	117.6	40.6	Landrace	
Xinyangyangyandou	ZDD03570	Henan	114	32.1	Landrace	
Runanpingdingshi	ZDD03222	Henan	114.4	33	Landrace	
Xiaolimoshidou	ZDD17767	Heilongjiang	126.4	45.4	Landrace	
Keshuang	ZDD00085	Heilongjiang	126.4	45.4	Landrace	
Mangcangjin	ZDD00078	Heilongjiang	126.4	45.4	Landrace	
Moheimoshidou	ZDD00332	Heilongjiang	126.6	51.7	Landrace	
Fengdihuang	ZDD00377	Jilin	126.6	43.9	Landrace	
Wuxiliuyueku	ZDD04403	Jiangsu	120.2	31.3	Landrace	
Donghaibaihuacaojia	ZDD03755	Jiangsu	118.8	34.5	Landrace	
Linhexiaofenqing	ZDD19929	Jiangsu	107.2	40.5	Landrace	
Pixiaodazihuacao	ZDD03739	Jiangsu	118	34.3	Landrace	
Tongshanpingdingwu	ZDD03901	Jiangsu	117.2	34.3	Landrace	

MOESM1 Soybean genotypes used for cloning and sequence diversity analysis of *Rhg4* （Continued）

**Name**	**Accession**	**Province**	**Longitude (° E)**	**Latitude (° N)**	**Population**	**Predigree**
Heidou	ZDD21846	Jiangxi	114.8	24.9	Landrace	
Mangdijin	ZDD00990	Liaoning	120.8	40.8	Landrace	
Tiejiaqing	ZDD07809	Liaoning	124.1	40.5	Landrace	
Bailudou	ZDD08728	Shanxi	113.6	37.3	Landrace	
Caihuangdou	ZDD09292	Shanxi	112.9	39.1	Landrace	
Chibuliuheidou	ZDD02258	Shanxi	111.8	38.9	Landrace	
Yinmanziheidou	ZDD02447	Shanxi	111.6	36.7	Landrace	
Yuanheidou	ZDD02481	Shanxi	112.5	40	Landrace	
Heidou	ZDD09566	Shanxi	111.2	35.1	Landrace	
Heidou	ZDD09351	Shanxi	111.8	38.1	Landrace	
Heigundou	ZDD09301	Shanxi	111.1	39	Landrace	
Huipizhiheidou	ZDD02315	Shanxi	111.1	38.5	Landrace	
Luoyeheidou	ZDD09226	Shanxi	112.4	39.3	Landrace	
Yuanboheidou	ZDD10261	Shaanxi	109.8	38.3	Landrace	
Heidou	ZDD10734	Shaanxi	108.9	34.6	Landrace	
Sangutiaoheidou	ZDD10254	Shaanxi	111.1	39	Landrace	
Chongmingbaimaobayuebai	ZDD05465	Shanghai	121.4	31.6	Landrace	
Zaojiaodou	ZDD13808	Sichuang	102.3	27.9	Landrace	
Dahuangmao	ZDD20709	Sichuang	102.3	27.9	Landrace	
Donghuangdou-1	ZDD13233	Sichuang	108.1	30	Landrace	
Meishanliuyuebao	ZDD12655	Sichuang	103.8	30.1	Landrace	
Taiwanerhao	ZDD17657	Taiwan	121.5	25	Landrace	
Yanqihuangdou	ZDD08124	Xinjiang	86.6	42.1	Landrace	
Dabaidou	ZDD17385	Yunnan	102.4	23.4	Landrace	
Jiuyueba	ZDD06233	Zhejiang	120.2	30.3	Landrace	
-	ZYD05183	Guangdong	113.3	24.8	*G.soja*	
-	ZYD05280	Guangxi	109.7	24	*G.soja*	
-	ZYD04734	Guizhou	108.1	27	*G.soja*	

MOESM1 Soybean genotypes used for cloning and sequence diversity analysis of *Rhg4* （Continued）

Name	Accession	Province	Longitude (° E)	Latitude (° N)	Population	Predigree
-	ZYD04754	Guizhou	108.4	28	*G.soja*	
-	ZYD02749	Hebei	119.2	39.7	*G.soja*	
-	ZYD03582	Henan	115	32.8	*G.soja*	
-	ZYD03632	Henan	114.9	33.4	*G.soja*	
-	ZYD00011	Heilongjiang	126.6	51.7	*G.soja*	
-	ZYD00037	Heilongjiang	127.5	50.2	*G.soja*	
-	ZYD00092	Heilongjiang	125.2	49.2	*G.soja*	
-	ZYD00099	Heilongjiang	130	48.9	*G.soja*	
-	ZYD00212	Heilongjiang	131.8	47.3	*G.soja*	
-	ZYD00257	Heilongjiang	134	46.8	*G.soja*	
-	ZYD00693	Heilongjiang	128	45.2	*G.soja*	
-	ZYD05289	Heilongjiang	126.4	45.4	*G.soja*	
-	ZYD04696	Hubei	119.2	39.7	*G.soja*	
-	ZYD00755	Jilin	122.5	45.4	*G.soja*	
-	ZYD01332	Jilin	130.4	42.9	*G.soja*	
-	ZYD01550	Jilin	125.7	40.9	*G.soja*	
-	ZYD01583	Jilin	128.2	41.4	*G.soja*	
-	ZYD04629	Jiangxi	118.3	28.7	*G.soja*	
-	ZYD02444	Liaoning	119.6	41.4	*G.soja*	
-	ZYD06156	Liaoning	123.9	42.3	*G.soja*	
-	ZYD05746	Inner Mongolia	123.5	48.1	*G.soja*	
-	ZYD02878	Shanxi	112.3	39	*G.soja*	
-	ZYD03149	Shanxi	110.7	35.6	*G.soja*	
-	ZYD03757	Shaanxi	109.1	35.1	*G.soja*	
-	ZYD04594	Tibet	97.5	28.7	*G.soja*	

[a] Plant introduction from the United States of America

[b] Plant introduction from Japan

[c] Plant introduction from France
