# Supplementary material for: DNA sequence polymorphism of the Rhg4 candidate gene conferring resistance to soybean cyst nematode in Chinese domesticated and wild soybeans
Source: Mol Breed. 2012 Feb 18;30(2):1155–62. doi: 10.1007/s11032-012-9703-1 (PMC3410032; doi:10.1007/s11032-012-9703-1)
Supplement: Supplementary file 2 — Primers designed for PCR amplification and sequencing (PDF 36 kb) [file 11032_2012_9703_MOESM2_ESM.pdf]

DNA sequence polymorphism of the *Rhg4* candidate gene conferring resistance to soybean cyst nematode in Chinese domesticated and wild soybeans

Moleculare Breeding

Cuiping Yuan · Yinghui Li · Zhangxiong Liu · Rongxia Guan · Ruzhen Chang · Lijuan Qiu
Corresponding author, E-mail: qiu_lijuan@263.net

MOESM2 Primers designed for PCR amplification and sequencing

Primer	Sequence（5’>>3’）	PCR product (bp)
305U18	TGGCAGCACACATAAATA	1415
1698L21	GACGGTTTGGAGGAAAGAAAG	
1500U18	TCGGGCTGGTCTGAAACA	1065
2540L25	GGGATTGCCATTGAGAAACAAAGT C	
2420U27	GCGGCGTGATGGTTGGAACTATGTTGT	1300
3695L25	AACAGCTATAACCACCAAATACGAG	
3561U22	GATGGGAAGTATTCTGTTGAGA	1247
4782L26	GGGTGCTTGATTGGGTTTGTGAGATG	
4643U19	CTCTTGTGGAGCAATGGAA	876
5497L19	AACCACCTCATCCAACTCA	
841L19§	ATGTTGCACTACTGGATAC	-
747U21§	GGTAATGGATTCAATTCCTTC	-
2042U21§	CGGTACCCTCCTCGTCCTCTC	-
1850L21§	TCGAGGTCGATTAGGTTAGAG	-
3305U20§	AAGGCTTTTGGTCTATGAGT	-
3083L20§	CCTCGCTGAAATTATTCGTC	-
4095U20§	GTTCTCTGTCGCAACTAATC	-
4342L19§	TTGTCACTCTTCCAGTAGC	-

§ Primer used only for sequencing
