# Supplementary material for: DNA sequence polymorphism of the Rhg4 candidate gene conferring resistance to soybean cyst nematode in Chinese domesticated and wild soybeans
Source: Mol Breed. 2012 Feb 18;30(2):1155–62. doi: 10.1007/s11032-012-9703-1 (PMC3410032; doi:10.1007/s11032-012-9703-1)
Supplement: Supplementary file 4 — Neighbor-joining phylogenetic tree of 25 soybean varieties from the analysis of the alignment of Rhg4 (PDF 20 kb) [file 11032_2012_9703_MOESM4_ESM.pdf]

DNA sequence polymorphism of the *Rhg4* candidate gene conferring resistance to soybean cyst nematode in Chinese domesticated and wild soybeans

Moleculare Breeding

Cuiping Yuan · Yinghui Li · Zhangxiong Liu · Rongxia Guan · Ruzhen Chang · Lijuan Qiu
Corresponding author, E-mail: qiu_lijuan@263.net

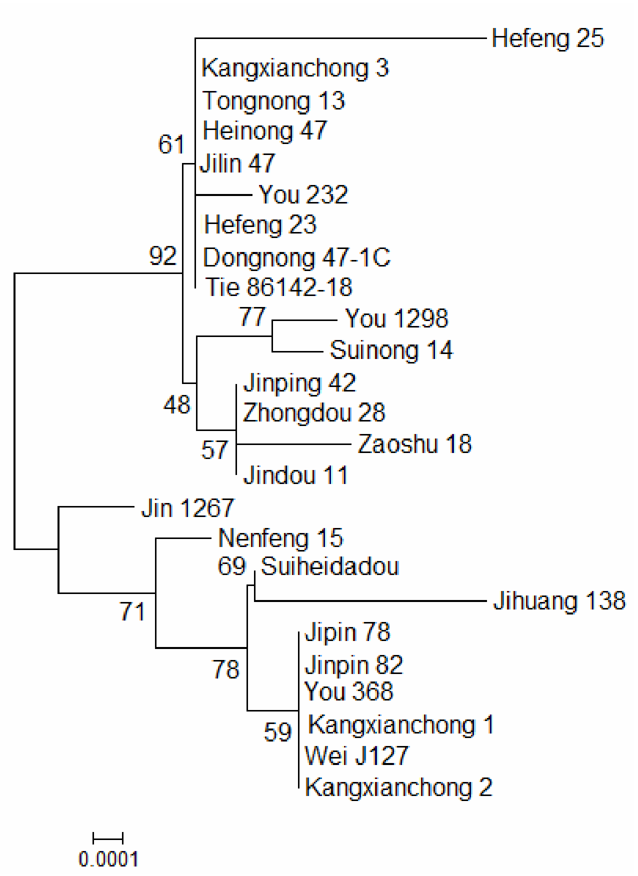

MOESM4 Neighbor-joining phylogenetic tree of 25 soybean varieties from the analysis of the alignment of *Rhg4*
